# Supplementary material for: Classification of Acute Myeloid Leukemia by Cell-Free DNA 5-Hydroxymethylcytosine
Source: Genes (Basel). 2023 May 28;14(6):1180. doi: 10.3390/genes14061180 (PMC10298116; doi:10.3390/genes14061180)
Supplement: Supplementary file 1 [file genes-14-01180-s001.zip › Supplementary_F_R_F.pdf]

Contents

Table S1. Characteristics of the acute myeloid leukemia patients. ………………………………..2

Table S2. List of differentially hydroxymethylated regions. ………………….……… Attachment

Table S3. Multivariate Cox regression analysis. ………………………………………………….3

Table S4. List of genes with differentially hydroxymethylated regions. …………….... Attachment

Table S5. Signaling pathways with differentially hydroxymethylated regions. ……… Attachment

Figure S1. Classification of acute myeloid leukemia patient samples. …………………………...4

Figure S2. Characteristics of cfDNA in acute myeloid leukemia patients. ………………………..5

Table S1. Characteristics of the acute myeloid leukemia patients.

	**Number** (N = 54)
**Sex (male/female)**	29/25
**Age, (median, range, y)**	
≥60 (66, 60-88)	33
<60 (48, 20-59)	21
**Treatment**	
No treatment	0
Chemotherapy	54
HSCT	31
No treatment info	0
**MFC, blasts in BM**	
Detected	33
Undetected	21
**Cytogenetics**	
Normal	28
Abnormal	25
Unknown	1
**Gene mutations** (positive/total no. of patients tested)	
TET2	4/54
IDH1	3/54
IDH2	3/54
WT1	3/54

HSCT, hematopoietic stem cell transplant; y, year; MFC, multiparameter flow cytometry; BM, bone marrow.

Table S3. Multivariate cox regression analysis.

Variable	Hazard ratio (95% CI)	*P*
Myeloblast ≤ 5%	4.6 (0.72-29.3)	0.11
Myeloblast > 5%	1.3 (0.17-10.0)	0.80
Cluster 2	1.0 (0.16-6.6)	0.98
Cluster 3	7.8 (1.1-53.3)	0.03

Myeloblast count of 0% and cluster 1 are reference groups in multivariate cox regression analysis.

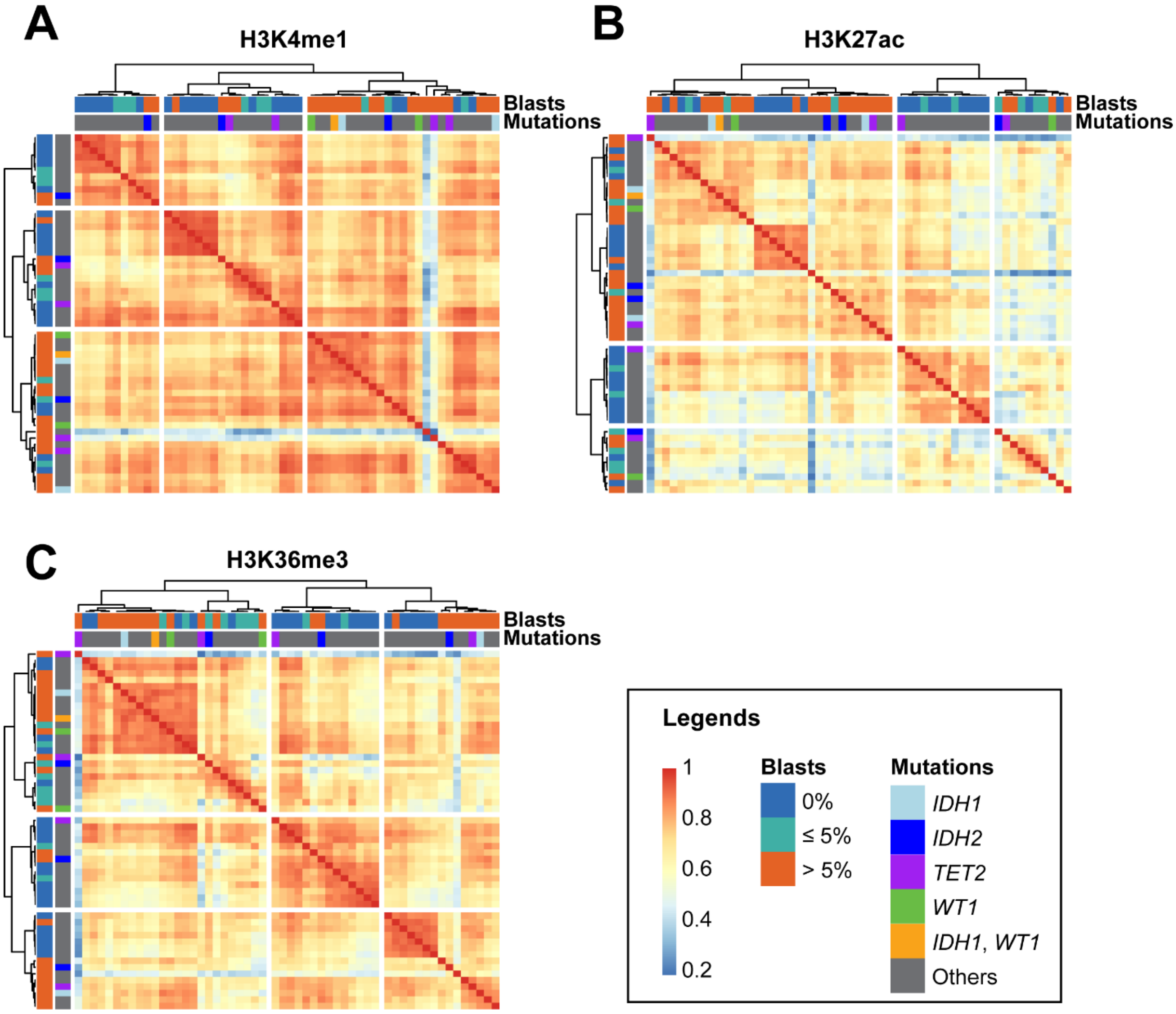

Figure S1. Classification of acute myeloid leukemia patient samples. Unsupervised hierarchical clustering of 54 AML samples using pairwise Pearson's correlation coefficient of 5-hydroxymethylcytosine (5hmC) levels in 500 most variable regions marked with H3K4me1 (A), H3K27ac (B), and H3K36me3 (C).

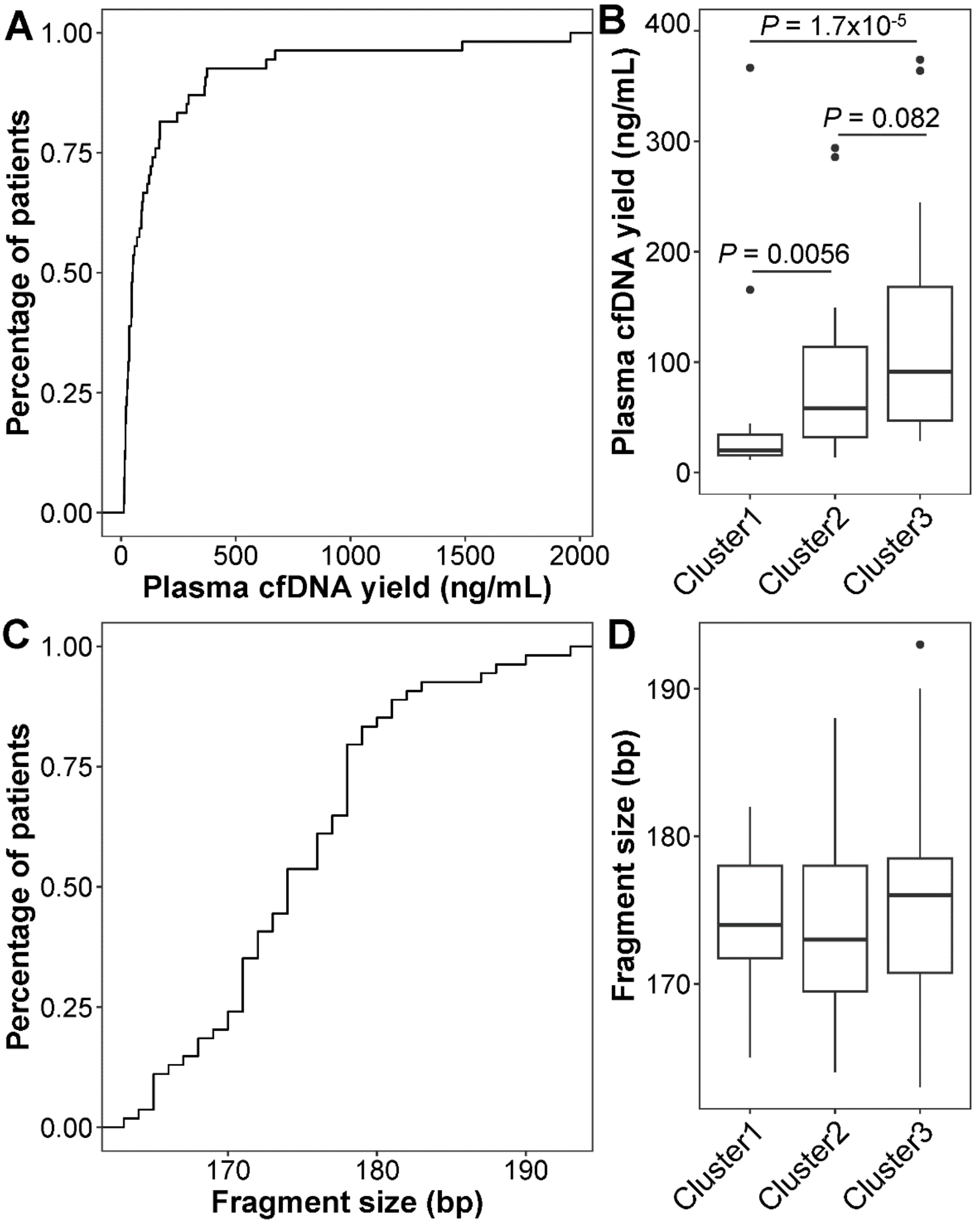

Figure S2. Characteristics of plasma cfDNA in acute myeloid leukemia patients. (A) Cumulative frequency distribution of plasma cfDNA yield in acute myeloid leukemia (AML) patients. (B) Boxplot of plasma cfDNA yield among three clusters. The center line represents median. Bounds of box represent 25th and 75th percentiles. Whiskers are Tukey whiskers. (C) Cumulative frequency distribution of plasma cfDNA fragment sizes in AML patients. (D) Boxplot of plasma cfDNA fragment sizes among three clusters. The center line represents median. Bounds of box represent 25th and 75th percentiles. Whiskers are Tukey whiskers.
